# TNFα aggravates detrimental effects of SARS-CoV-2 infection in the liver

**DOI:** 10.3389/fimmu.2023.1151937

**Published:** 2023-03-31

**Authors:** Jöran Lücke, Mikolaj Nawrocki, Josa Schnell, Nicholas Meins, Fabian Heinrich, Tao Zhang, Franziska Bertram, Morsal Sabihi, Marius Böttcher, Tom Blankenburg, Marie Pfaff, Sara Notz, Jan Kempski, Matthias Reeh, Stefan Wolter, Oliver Mann, Jakob R. Izbicki, Marc Lütgehetmann, Anna Duprée, Anastasios D. Giannou, Benjamin Ondruschka, Samuel Huber

**Affiliations:** ^1^ Section of Molecular Immunology and Gastroenterology, I. Department of Medicine, University Medical Center Hamburg-Eppendorf, Hamburg, Germany; ^2^ Hamburg Center for Translational Immunology (HCTI), University Medical Center Hamburg-Eppendorf, Hamburg, Germany; ^3^ Department of General, Visceral and Thoracic Surgery, University Medical Center Hamburg-Eppendorf, Hamburg, Germany; ^4^ I. Department of Medicine, University Medical Center Hamburg-Eppendorf, Hamburg, Germany; ^5^ Institute of Legal Medicine, University Medical Center Hamburg-Eppendorf, Hamburg, Germany; ^6^ Institute of Medical Microbiology, Virology, and Hygiene, University Medical Center Hamburg-Eppendorf, Hamburg, Germany; ^7^ The Calcium Signaling Group, Department of Biochemistry and Molecular Cell Biology, University Medical Center Hamburg-Eppendorf, Hamburg, Germany

**Keywords:** SARS-CoV-2, liver, TNFa, post-mortem, Cxcl3, Cxcl8, Icam1

## Abstract

Coronavirus disease 2019 (COVID-19) is caused by the severe acute respiratory syndrome coronavirus 2 (SARS-CoV-2). This virus does not only lead to pulmonary infection but can also infect other organs such as the gut, the kidney, or the liver. Recent studies confirmed that severe cases of COVID-19 are often associated with liver damage and liver failure, as well as the systemic upregulation of pro-inflammatory cytokines such as tumor necrosis factor-alpha (TNFα). However, the impact these immune mediators in the liver have on patient survival during SARS-CoV-2 infection is currently unknown. Here, by performing a post-mortem analysis of 45 patients that died from a SARS-CoV-2 infection, we find that an increased expression of *TNFA* in the liver is associated with elevated mortality. Using publicly available single-cell sequencing datasets, we determined that Kupffer cells and monocytes are the main sources of this TNFα production. Further analysis revealed that TNFα signaling led to the upregulation of pro-inflammatory genes that are associated with an unfavorable outcome. Moreover, high levels of *TNFA* in the liver were associated with lower levels of interferon alpha and interferon beta. Thus, TNFα signaling in the infected SARS-CoV-2 liver correlates with reduced interferon levels and overall survival time.

## Introduction

Since the start of the coronavirus disease 2019 (COVID-19) pandemic at the end of 2019, infection with the severe acute respiratory syndrome coronavirus 2 (SARS-CoV-2) has claimed millions of lives (1). Although the successful development of multiple vaccines generally slowed the spreading and mortality of SARS-CoV-2 infection (2, 3), the infection still poses major challenges to physicians and researchers alike (4). One realization of the extended research committed to COVID-19 is that SARS-CoV-2 is also capable of infecting organs other than the respiratory tract. For example, SARS-CoV-2 infections in the kidney (5), the heart (6), the gut (7, 8), or the liver (9) and more have been reported.

Early on, it was reported that the main entry protein for SARS-CoV-2, angiotensin-converting enzyme 2 (ACE2), is also expressed in the liver, mainly in cholangiocytes and hepatocytes (10, 11). Indeed, the generation of ACE2 and transmembrane protease serine 2 (TMPRSS2)-expressing human liver organoids permitted SARS-CoV-2 to infect these cells (12). Further studies revealed that SARS-CoV-2 could be detected in post-mortem samples of livers (13–16). Moreover, histopathological changes in the way of microscopic fibrosis and steatosis, infiltration of lymphocytes, cell necrosis, or microthrombosis were described (17). In line with these findings, clinical signs of acute liver injury are a common feature of COVID-19 infection (18–23). Likewise, aggravated cases of SARS-CoV-2 infection are often associated with eliciting a cytokine storm, which is described as an increase in pro-inflammatory mediators such as interleukin (IL)-1β, IL-6, IL-8 or tumor necrosis factor-alpha (TNFα) (24). In line with this finding, elevated levels of these cytokines in the serum of patients are associated with reduced survival (25). However, how hepatic SARS-CoV-2 infections influence the local and systemic immune response and might relate to overall patient survival is currently unknown.

TNFα is a pro-inflammatory cytokine, that – although initially being thought of as mainly secreted by macrophages and monocytes (26, 27) - can be produced by many innate and adaptive immune cells (28). It can signal through one of two receptors, namely tumor necrosis factor receptor (TNFR)1 and TNFR2 (29). Downstream effects include the activation of the nuclear factor ‘kappa-light-chain-enhancer’ of activated B-cells (NF-κB) pathway, the mitogen-activated protein kinase (MAPK) pathways, and induction of apoptosis due to promoting cleavage of caspase 8 (29). On the one hand, TNFα is a powerful inductor of acute phase proteins and thus, aids the host in defending against pathogens such as tuberculosis (30). On the other hand, the last decade provided mounting evidence of its pathogenic role in maintaining autoimmune diseases such as inflammatory bowel disease or rheumatoid arthritis (31). Hence, a therapeutical blockade of this cytokine is nowadays a pivotal pillar for treating these diseases (31). Interestingly, these TNFα-antagonists were also successfully evaluated as a treatment option in severe cases of COVID-19 (32). However, despite the effectiveness of their usage, the pathological mechanisms of these cytokines during COVID-19 are still not fully understood.

Here, using a post-mortem analysis of patients that died from COVID-19, we discover for the first time that elevated RNA levels of *TNFA* in the liver, but not in the blood, are connected to decreased survival. Further analysis reveals that monocytes, as well as Kupffer cells, comprise the main source of TNFα during SARS-CoV-2 infection. We further found that TNFα signaling was associated with increased levels of genes in immune cells that were connected to an unfavorable immune response, such as *ICAM1* and *CXCL8*. Moreover, increased TNFα signaling in hepatocytes of COVID-19-infected specimens was associated with increased production of acute-phase proteins such as *SAA1* and *SERPINA1*. In our patient cohort, high levels of liver-derived *TNFA* further inversely correlated with *IFNA* and *IFNB*. We also found that SARS-CoV-2 detectability in the liver was associated with a reduced survival time. In summary, liver-derived TNFα during SARS-CoV-2 infection correlates to reduced interferon levels and reduced survival time.

## Methods

### Autopsies and collection of clinical data

The data and sample collection were performed in the timespan between April 2020 and April 2021. In total, 45 patients that died from COVID-19 were admitted to the Institute of Legal Medicine and were subsequently included in the study by full autopsies. Initially, a reverse transcription-quantitative polymerase chain reaction from nasopharyngeal swab samples was performed as part of the routine diagnostic at the Institute of Microbiology, Virology, and Hygiene for a first assessment. Then, the cause of death was determined in accordance with the current literature (33). Patients with other causes of death than SARS-CoV-2 were excluded from the analysis. Corpses with advanced putrefactive changes were excluded also. All bodies were stored at 4°C upon admission to the Institute of Legal Medicine. All subsequent autopsies were performed at the Legal Medicine morgue following the German Society for Forensic Medicine guidelines. Tissue samples were harvested as triplicates from the liver, the duodenum, and blood and were frozen in liquid nitrogen. Comprehensive clinical data were acquired post-mortem from different sources. The informed consent of relatives or legal representatives was obtained. The study was approved by the Ethics Committee of the Hamburg Chamber of Physicians (reference numbers PV7311 and 2020-10353-BO-ff) and conducted according to the guidelines of Helsinki.

### Quantitative RT-PCR for cytokines

Total RNA from tissues was extracted using the Rneasy^®^ Plus Mini Kit (Qiagen, Hilden, Germany) according to the manufacturer’s instructions. After adjusting the RNA yield, the high-capacity cDNA synthesis Kit (Applied Biosystems, Waltham, US) was used for cDNA synthesis. Real-time PCR was performed using the Kapa Probe Fast qPCR Master Mix (Kapa Biosystems, Wilmington, US) on the StepOne Plus system (Applied Biosystems). Probes were purchased from Applied Biosystems (**Table 1**). The relative expression was normalized to the housekeeping gene, HPRT, and was then calculated using the 2-ΔΔCt method.

**Table 1:** Probes used for human RNA quantification with RT-qPCR.

Gene	Probe name
*HPRT1*	Hs02800695_m1
*IFNA2*	Hs00265051_s1
*IFNB1*	Hs01077958_s1
*TNFA*	Hs01113624_g1

### Quantitative RT-PCR for SARS-CoV-2

The protocol was carried out as previously reported (34). The tissue samples were transferred to 2ml tubes filled with ceramic beads (Precellys Lysing Kit) and PBS and were subsequently homogenized (Precellys 24, Bertin, Rockville, US). Then, 200μl of this lysate was used for further extraction with MagnaPure96 (Roche, Mannheim, Germany). Primer (5'-ACAGGTACGTTAATAGTTAATAGCmGT-3’, 400nM end concentration; 5' TATTGCAGCAGTACGCACAmCA-3', 400nM end concentration) and probe (5'-Fam- ACACTAGCC/ZEN/ATCCTTACTGCGCTTCG-Iowa Black FQ-3', 100nM end concentration) were used which were acquired by Integrated DNA Technologies (IDT, Leuven, Belgium). One-step RT-PCR (25μl volume) was performed by the LightCycler480 system (Roche) with a one-step RNA control kit (Roche) using the master mix provided and 5μl of the eluate. The Ct value for the target SARS-CoV-2 RNA was measured by taking advantage of the second derivative maximum method. Standard RNA reference material (obtained from INSTAND e.V., Düsseldorf, Germany) was used for correct quantification. Quantitative β-globin PCR was carried out with the respective TaqMan primer set (Thermo-Fischer, 401846) and the DNA control kit (Roche). Samples were run on the LightCycler480 system. SARS-CoV-2 RNA levels in tissues were then normalized to β-globin DNA.

### Single-cell sequencing

For the pre-processing methods of single-cell sequencing data, we refer to Delorey et al. (35). Processed sequencing data (sc/snRNA-Seq and bulk) was obtained from the Gene Expression Omnibus (GEO, https://www.ncbi.nlm.nih.gov/geo/) under accession number GSE171668. Cell annotation from the source data was adopted. The gene expression tables corresponding to liver samples were integrated with harmony (harmony v0.1.0, Seurat v 4.2.0). Umaps were created using Seurat. Curated gene Signatures were obtained from MSigDB - GSEA and the enrichment was calculated with Seurat. For the pseudobulk differential expression analysis of hepatocytes, the cells were divided into high or low *TNFA* responders depending on the signature score of the *TNFA* up-regulation signature. In the next step, the cells were aggregated per sample and condition. For the analysis of immune cells, the immune cells were additionally aggregated. The differential gene expression analysis was performed using the DESeq2 package (v 1.36.0). Gene Ontology enrichment analysis was performed with ClusterProfiler (version 4.4.4). Then, the differentially expressed genes with adjusted p value < 0.05 were selected.

### Statistics

The analysis of categorical variables was made by Fisher’s exact or Chi-square test, while continuous variables were compared using the Mann-Whitney U-test. Correlation coefficients were estimated using a correlation between pairs of variables, using pairwise deletion of observation in case of missing values by default. Bonferroni adjusted significance levels of correlation coefficients are given. Survival function estimates were calculated using the Kaplan-Meier method and were subsequently analyzed using the log-rank test. P-values equal to or less than 0.05 were considered statistically significant. The statistical analysis was done with STATA/MP, Version 17.0 (StataCorp, Texas, USA). GraphPad Prism software version 9.1.1 (GraphPad Software, CA, USA) was used for data presentation. Graphical illustrations were created using biorender.com.

## Results

### Elevated levels of *TNFA* in the liver are associated with a decreased survival time

**Figure 1:**
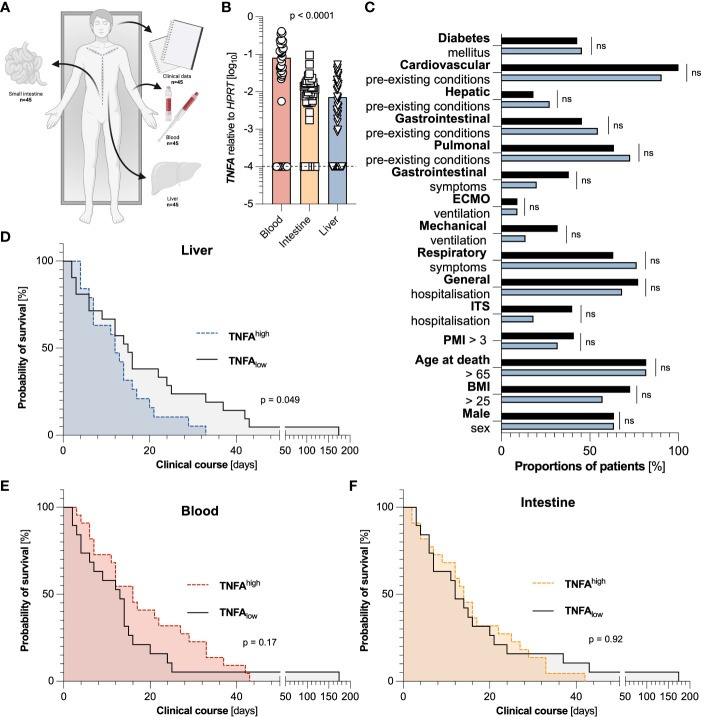
Elevated levels of *TNFA* in the liver, but not in the blood or small intestine are associated with a decreased survival time. **(A)** Representative scheme of the experimental setup in which clinical data and tissue samples of blood, liver, and duodenum of 45 individuals that died from COVID-19 were collected. **(B)** Relative expression of *TNFA* mRNA in the blood (left, red, n=44), the intestine (middle, yellow, n=45), and the liver (right, blue, n=44), as measured by reverse transcriptase polymerase chain reaction (RT-PCR). One blood sample and one liver sample had to be excluded due to undetectable *HPRT* values. The dashed black line represents the limits of detection. Statistical analysis was performed using one-way ANOVA. **(C)** Cohort characteristics of patients with low (below the median) hepatic expression of *TNFA* (n=22, black), and high (above the median) hepatic expression of *TNFA* (n=22, blue) are depicted. **(D)** Kaplan Meier analysis of survival length in patient cohorts divided according to low (below the median) hepatic expression of *TNFA* (n=22, black), and high (above the median) hepatic expression of *TNFA* (n=22, blue) in the liver. **(E)** Kaplan Meier analysis of survival length in patient cohorts divided according to low (below the median) systemic expression of *TNFA* (n=22, black), and high (above the median) systemic expression of *TNFA* (n=22, red) in the blood. **(F)** Kaplan Meier analysis of survival length in patient cohorts divided according to low (below the median) intestinal expression of *TNFA* (n=22, black), and high (above the median) intestinal expression of *TNFA* (n=23, yellow) in the small intestine. Horizontal lines represent means ± SEM; each symbol indicates one sample from one patient. ns, not significant.

We aimed to investigate the role of liver-specific TNFα-based immune response during SARS-CoV-2 infection. To this end, we acquired autopsy samples from blood, duodenum, and liver from 45 individuals that died from COVID-19 (**Figure 1A**). When comparing expression levels between different organs, we found that *TNFA* was highest expressed in blood, while being expressed at significantly lower levels in the duodenum, and even lower in liver tissue (**Figure 1B**).

To then examine the association of TNFα in different organs on the overall survival time, we divided the cohorts according to the median of *TNFA* expression in the liver, the blood, and the intestine, respectively. Interestingly, elevated expression of *TNFA* in the liver (**Figure 1D**), but not in the blood (**Figure 1E**) or small intestine (**Figure 1F**), was significantly associated with a reduced survival time, or more precisely, the time between the first positive SARS-CoV-2 test and death. Of note, in the liver, these two groups did not display significant differences in all investigated parameters or comorbidities, including sex, BMI, age, post-mortal interval (PMI), and hospitalization (**Figure 1C**). Likewise, in the groups divided according to the median of *TNFA* expression in blood and intestine, no significant difference could be detected in population characteristics except for the division of sex and hepatic pre-existing conditions in the cohorts of blood and intestine, respectively (**Figure S1**). In conclusion, elevated levels of *TNFA* in the liver are associated with a decreased survival time.

### Liver-derived *TNFA* is predominantly produced by Kupffer cells and monocytes during SARS-CoV-2 infection

**Figure 2:**
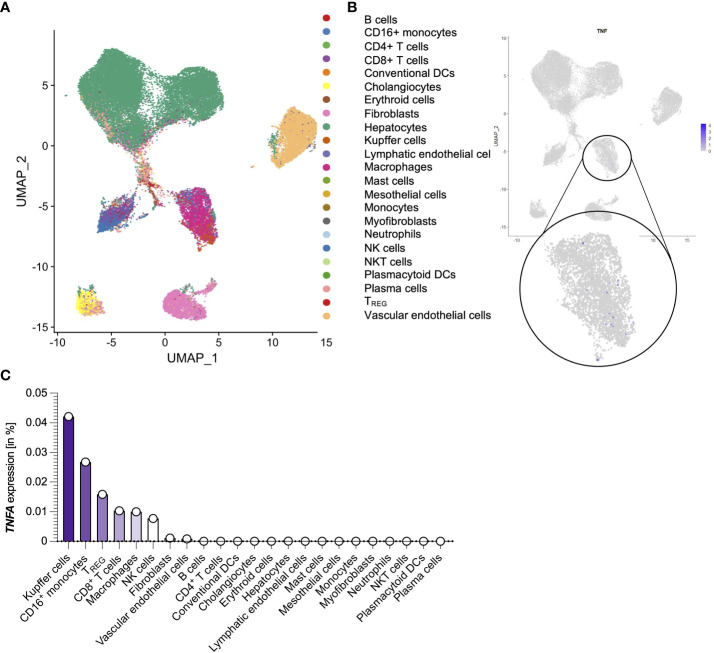
Liver-derived TNFA is predominantly produced by Kupffer cells and monocytes during SARS-CoV-2 infection. **(A)** Cell clusters from the livers of 15 SARS-CoV-2 infected patients that were identified using single-cell sequencing of a publicly available dataset (35). **(B)** *TNFA* expression of the cell clusters as described in **(A)**. **(C)** Percentage of *TNFA*-expressing cells divided by clusters as described in **(A)**. ns, not significant.

Next, we wanted to determine the source of TNFα in the liver of the SARS-CoV-2 infected deceased. To that end, we took advantage of a publicly available dataset consisting of 15 autopsy samples from the livers of recently deceased SARS-CoV-2 infected patients (**Figure 2A**) (35). When analyzing the expression of *TNFA* within this dataset, only a fraction of cells in the liver had detectable *TNFA* expression levels (**Figure 2B**). Further analysis revealed that Kupffer cells were the predominant producers of *TNFA* within the livers of SARS-Cov-2 deceased patients, followed by monocytes, T cells, and macrophages (**Figure 2C**). In summary, Kupffer cells and monocytes are the major producers of TNFα in the liver during SARS-CoV-2 infection.

### *TNFA*-responsive hepatocytes and immune cells are transcriptionally distinct

**Figure 3:**
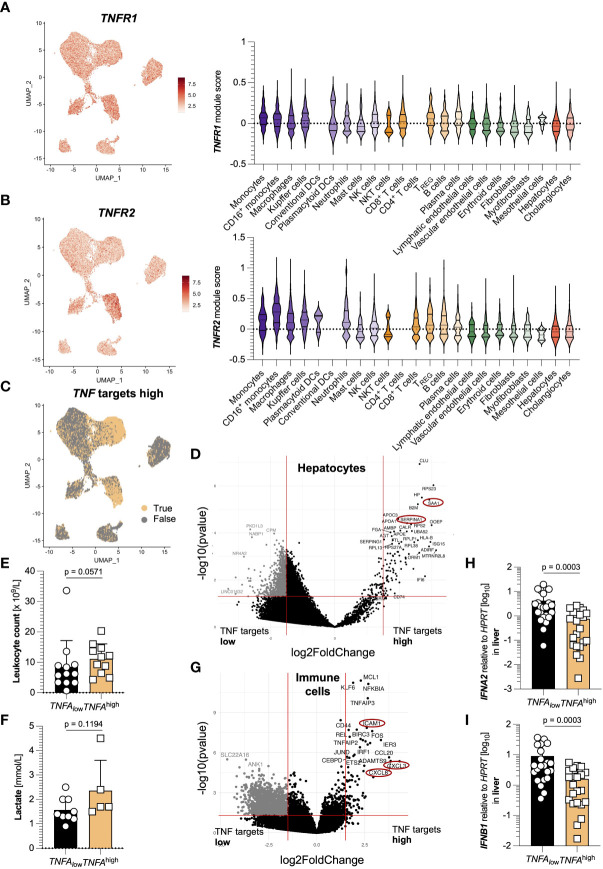
*TNFA*-responsive hepatocytes and immune cells are transcriptionally distinct. **(A)** Left: *TNFR1* expression in cell clusters from the livers of 15 SARS-CoV-2 infected patients identified using single-cell sequencing; right: *TNFR1* expression divided by cell cluster. **(B)** Left: *TNFR2* expression in cell clusters from **(A)**; right: *TNFR2* expression divided by cell cluster. **(C)** Elevated expression of *TNFA*-associated target genes in clusters from **(A)**. **(D)** Volcano plot highlighting the differences in the clusters of hepatocytes from **(A)** between elevated and non-elevated expression of *TNFA*-associated target genes. **(E, F)** First available levels of **(E)** leucocyte count (x10^9^/L, n=23) and **(F)** lactate (in mmol/L, n=14) upon SARS-CoV-2 diagnosis, determined by screening of patient´s laboratory findings, divided according to low (below the median of all 44 patients, black) and high (above the median of all 44 patients, yellow) expression of hepatic *TNFA*. Statistics by Mann-Whitney test. **(G)** Volcano plot highlighting the differences in the clusters of immune cells from **(A)** between elevated and non-elevated expression of *TNFA*-associated target genes. **(H, I)** Relative expression of **(H)** *IFNA2* and **(I)** *IFNB1* in the liver of patients divided according to low (below the median, n=22, black) and high (above the median, n=22, yellow) relative expression of *TNFA* mRNA in the liver. Statistics by Mann-Whitney test. Horizontal lines represent means ± SEM; each symbol indicates one sample from one patient. ns, not significant.

In the next step, we assessed potential target cells of TNFα-signaling, for which we first analyzed the expression of the TNFα receptors, TNFR1 and TNFR2. In the liver of SARS-CoV-2 infected, *TNFR1* was expressed ubiquitously, including hepatocytes and immune cells (**Figure 3A**), while *TNFR2* was mainly expressed in hematopoietic cells (**Figure 3B**). When investigating the upregulation of TNFα-associated pathways, we found that hepatocytes with such activated pathways formed a distinct sub-cluster (**Figure 3C**). Moreover, the majority of immune cells also responded to TNFα. Further analysis revealed a significantly different transcriptional profile of hepatocytes with upregulated TNFα-associated pathways: For example, acute phase proteins such as *SAA1* and *SERPINA1* were significantly upregulated in hepatocytes with high activation of TNFα-associated pathways (**Figure 3D**). This prompted us to investigate the systemic immune response in the patients of the *TNFA* ^high^ and *TNFA*_low_ groups. Indeed, leucocyte count and lactate, general markers for severe inflammation, hemolysis, and ultimate organ failure, were upregulated in patients with increased hepatic *TNFA* expression by trend (**Figures 3E, F**).

In immune cells, the activation of TNFα-associated pathways led to an increased expression of genes such as *CXCL3*, *CXCL8*, and *ICAM1* (**Figure 3G**). Furthermore, *IFNA2* and *IFNB1* were significantly downregulated in patients with increased *TNFA* expression (**Figures 3H, I**). Of note, we also performed GO enrichment analysis of hepatocytes and immune cells with activated TNFa-associated pathways, and found pathways related to antigen processing and presentation enriched in hepatocytes, while immune cells showed pathways related to TNF signaling (**Figure S2**). Taken together, TNFα-signaling in hepatocytes and immune cells leads to distinct transcriptional changes that might have systemic consequences.

### Intrahepatic SARS-CoV-2 detection is an independent factor for decreased survival time

**Figure 4:**
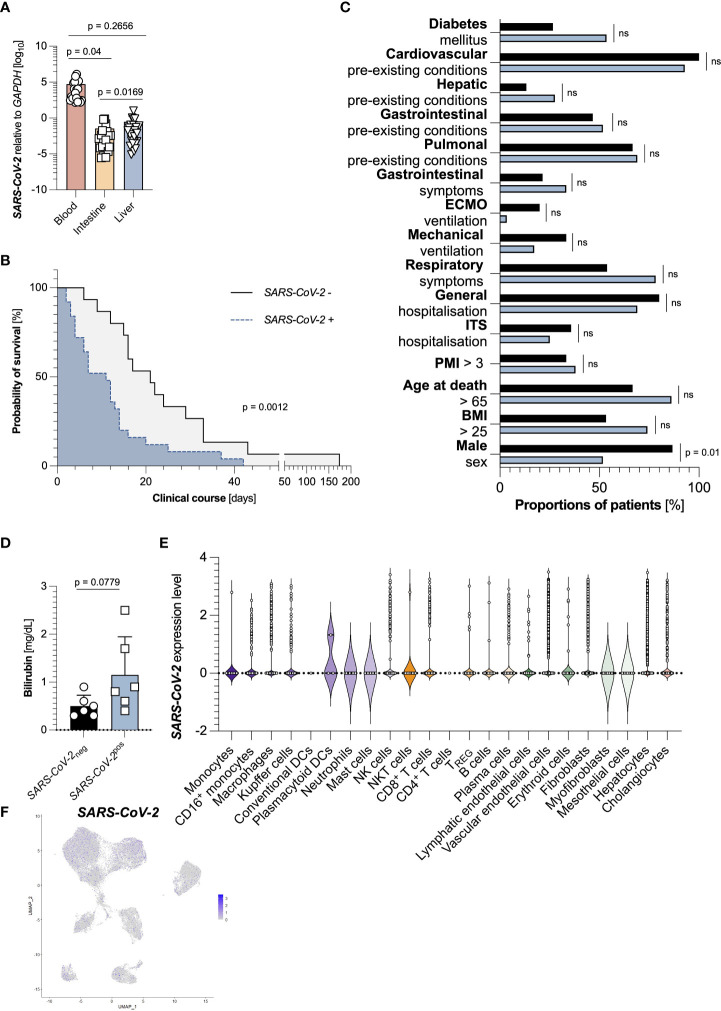
Intrahepatic SARS-CoV-2 detection is an independent factor for decreased survival time. **(A)** Relative expression of SARS-CoV-2 to *GAPDH* in the blood (left, red, n=44), the intestine (middle, yellow, n=45), and the liver (right, blue, n=44), as measured by RT-PCR. **(B)** Kaplan Meier analysis of survival length in patient cohorts divided according to an undetectable hepatic expression of SARS-CoV-2 (n=15, black), and a detectable hepatic expression of SARS-CoV-2 (n=29, blue) in the liver. **(C)** Cohort characteristics of patients with undetectable hepatic expression of SARS-CoV-2 (n=15, black), and detectable hepatic expression of SARS-CoV-2 (n=29, blue) in the liver. **(D)** First available levels of bilirubin (mg/dL, n=12) upon SARS-CoV-2 diagnosis, determined by screening of patient´s laboratory findings, divided according to undetectable (black, left, n=6) and detectable (blue, right, n=6) hepatic SARS-CoV-2 expression. Statistics by Mann-Whitney test. **(E)** SARS-CoV-2 expression divided by cell cluster from the livers of 15 SARS-CoV-2 infected patients. **(F)** SARS-CoV-2 expression in cell clusters from the livers of 15 SARS-CoV-2 infected patients identified using single-cell sequencing. Horizontal lines represent means ± SEM; each symbol indicates one sample from one patient. ns, not significant.

Finally, we wondered whether the detection of *SARS-CoV-2* RNA in the liver itself would also be associated with decreased survival, as is the case for many other organs. Although viral loads of *SARS-CoV-2* in the blood were the highest, it could be detected in many samples of duodenum and liver as well (**Figure 4A**). Indeed, the detectability of *SARS-CoV-2* RNA in the liver was associated with a significantly decreased survival time (**Figure 4B**). These groups did not significantly differ in population characteristics except for the division in sex (**Figure 4C**).

However, levels of bilirubin, a surrogate marker for liver function and failure, were increased in patients with detectable hepatic *SARS-CoV-2* RNA by trend (**Figure 4D**). We finally asked which cells within the livers could be infected by *SARS-CoV-2*. Returning to the publicly available dataset, we found that multiple subsets contained *SARS-CoV-2* positive cells. Interestingly, many *SARS-CoV-2* positive cells were hepatocytes (**Figures 4E, F**). However, we did not detect any significantly expressed genes between these cells (data not shown). In summary, SARS-CoV-2 detection in the liver is connected to a decreased survival time.

## Discussion

Despite the worldwide implementation of vaccination strategies against SARS-CoV-2 infections with the turn of the year to 2021, COVID-19 still possesses a significant threat to patients (4, 36), requiring continuous research on routes of infections and disease-enhancing factors. Although primarily an infection of the lung, SARS-CoV-2 can also infect other organs such as the liver (9). While a dysregulated systemic immune system is a common feature of severe COVID-19 (37), only a few observations were made regarding local immune responses in peripheral organs affected by SARS-CoV-2 infection.

Here, we show that an elevated *TNFA* expression in the liver, but not in the blood or the small intestine, is associated with a reduced time until death. First, the finding that *TNFA* expression in the blood is not associated with the survival period in our cohort is, to some extent surprising, since it seemingly contradicts previous reports (25). However, we did not measure TNFα levels in the serum *via* ELISA but rather assessed the *TNFA* RNA expression in the blood, which primarily consists of the transcriptome of blood cells, foremost leucocytes. Moreover, our cohort consists only of patients that died from SARS-CoV-2 infection and had a lethal course of this disease, which distinguishes our cohort from others that were investigated elsewhere.

Overall, the pathogenic role of TNFα in other hepatic diseases such as non-alcoholic fatty liver disease (38–40) or autoimmune liver disease (41, 42) is well-established. This effect is often associated with the capability of TNFα to induce apoptosis and necroptosis of hepatocytes (43, 44). Interestingly, in our patient cohort, high *TNFA* expression in the liver was neither associated with an increased local viral load nor with elevated laboratory markers for liver damage (data not shown). However, we found that an increased *TNFA* expression in the liver was associated with elevated systemic inflammation markers by trend. Using publicly available single cells sequencing resources, we found that upregulated TNFα-pathways in the liver were associated with increased *SAA1* and *SERPINA1* expression. Indeed, a high systemic expression of these two acute phase proteins is associated with severe and fatal COVID-19 courses (45, 46). We also found that immune cells with upregulated TNFα-pathways expressed increased *CXCL3*, *CXCL8*, and *ICAM1* genes, which are equally known to be associated with a detrimental outcome of SARS-CoV-2 infection (47, 48). These two observations might explain the pathogenic role of TNFα-signalling in the liver upon SARS-CoV-2 infection. Nonetheless, precise molecular mechanisms explaining this observation are currently lacking and require further investigation.

Finally, we found a negative correlation between the detectability of SARS-CoV-2 in the liver and the time of survival in our patient cohort. While it is published that this virus can infect the liver (13–16) and that liver damage is a common feature of SARS-CoV-2 infections (49), no direct associations between the viral load in the liver and survival have been drawn so far. However, whether infection of the liver is the cause of reduced survival time, or whether a detectable SARS-CoV-2 load in the liver is just a sign of an exceptionally aggravated course of the disease is currently unknown.

Despite all strengths of this study, there are some limitations as well that deserve being discussed. First, livers were not perfused post-mortem, so that a contamination of SARS-CoV-2 and cytokine expression levels from the blood itself cannot be excluded. Second, patient groups were divided according to *TNFA* RNA expression in different organs at the time of death, disregarding previous dynamics of *TNFA* RNA expression prior to their death. Finally, the analysis was carried out investigating RNA expression, that sometimes undergoes heavy posttranscriptional modifications and does not necessarily translate to protein expression in all cases.

Taken together, our data present a connection between elevated *TNFA* expression in the liver and a reduced survival time. We found that in SARS-CoV-2 infected livers, TNFα was mainly produced by monocytes and Kupffer cells. *TNFA*-associated pathways were mainly upregulated in hepatocytes and immune cells, while the activation of a *TNFA* pathway in hepatocytes was associated with an upregulation of acute phase proteins, while its activation in immune cells led to an increase of genes such as *CXCL3*, *CXCL8*, and *ICAM1*. In conclusion, these findings highlight a previously unrecognized role of hepatic TNFα during fatal SARS-CoV-2 infection.

## Data availability statement

The raw data supporting the conclusions of this article will be made available by the authors, without undue reservation.

## Ethics statement

The studies involving human participants were reviewed and approved by the Ethics Committee of the Hamburg Chamber of Physicians (reference numbers PV7311 and 2020-10353-BO-ff) and conducted according to the guidelines of Helsinki. Written informed consent to participate in this study was provided by the participants’ legal guardian/next of kin.

## Author contributions 

JL and MN designed all experiments and analyzed data. JL wrote the manuscript. MN performed all single-cell sequencing analysis. JS and NM performed RNA extraction and qPCR assays. FH, TZ, FB, MS, MB, TB, MP, SN, JK, MR, SW, OM, JI and AD provided critical intellectual input and edited the paper. ML performed measurements of SARS-CoV-2 levels, provided critical intellectual input and edited the paper. AG, BO, and SH conceived the idea and supervised the study. All authors contributed to the article and approved the submitted version.

## Supplementary material

Supplementary Figure 1 Cohort characteristics of *TNFA* ^high^ and *TNFA*_low_ groups in the blood and the intestine. **(A)** Cohort characteristics of patients with low (below the median) systemic expression of *TNFA* (n=22, black), and high (above the median) systemic expression of *TNFA* (n=22, red) in the blood are depicted. **(B)** Cohort characteristics of patients with low (below the median) intestinal expression of *TNFA* (n=22, black), and high (above the median) intestinal expression of *TNFA* (n=23, yellow) in the intestine are depicted.

Supplementary Figure 2 GO enrichment analysis of hepatocytes and immune cells with increased *TNFA*-associated target genes. **(A)** GO enrichment analysis in hepatocytes with elevated expression of *TNFA*-associated target genes from the livers of 15 SARS-CoV-2 infected deceased identified using single-cell sequencing. **(B)** GO enrichment analysis in immune cells with elevated expression of *TNFA*-associated target genes from the livers of 15 SARS-CoV-2 infected deceased identified using single-cell sequencing.
