# Supplementary figures and images for: TNFα aggravates detrimental effects of SARS-CoV-2 infection in the liver
Source: Front Immunol. 2023 Mar 31;14:1151937. doi: 10.3389/fimmu.2023.1151937 (PMC10102423; doi:10.3389/fimmu.2023.1151937)

# Supplementary figure 1

a

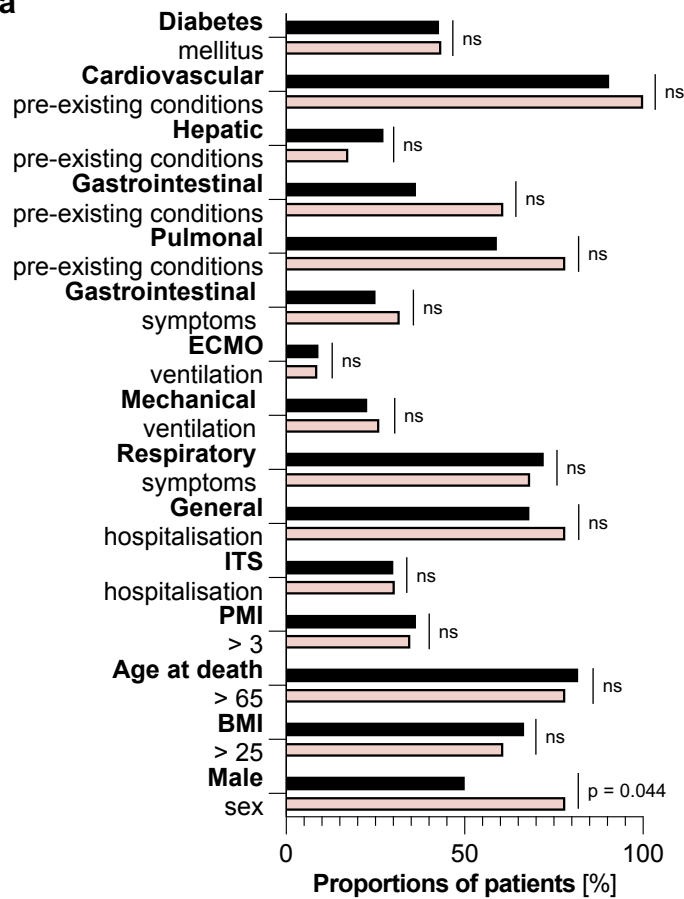

b

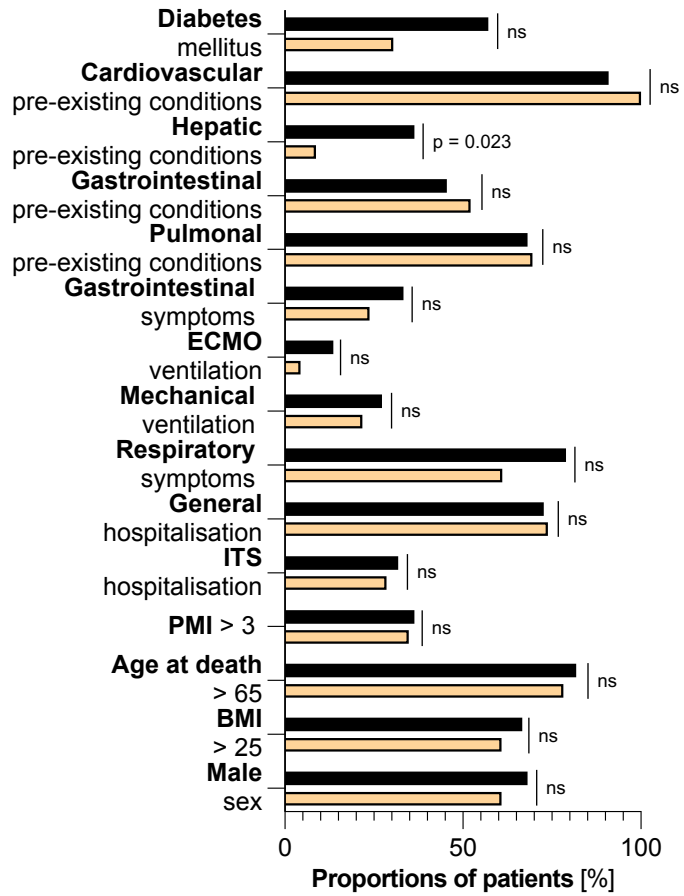

# Supplementary figure 2

## a Hepatocytes

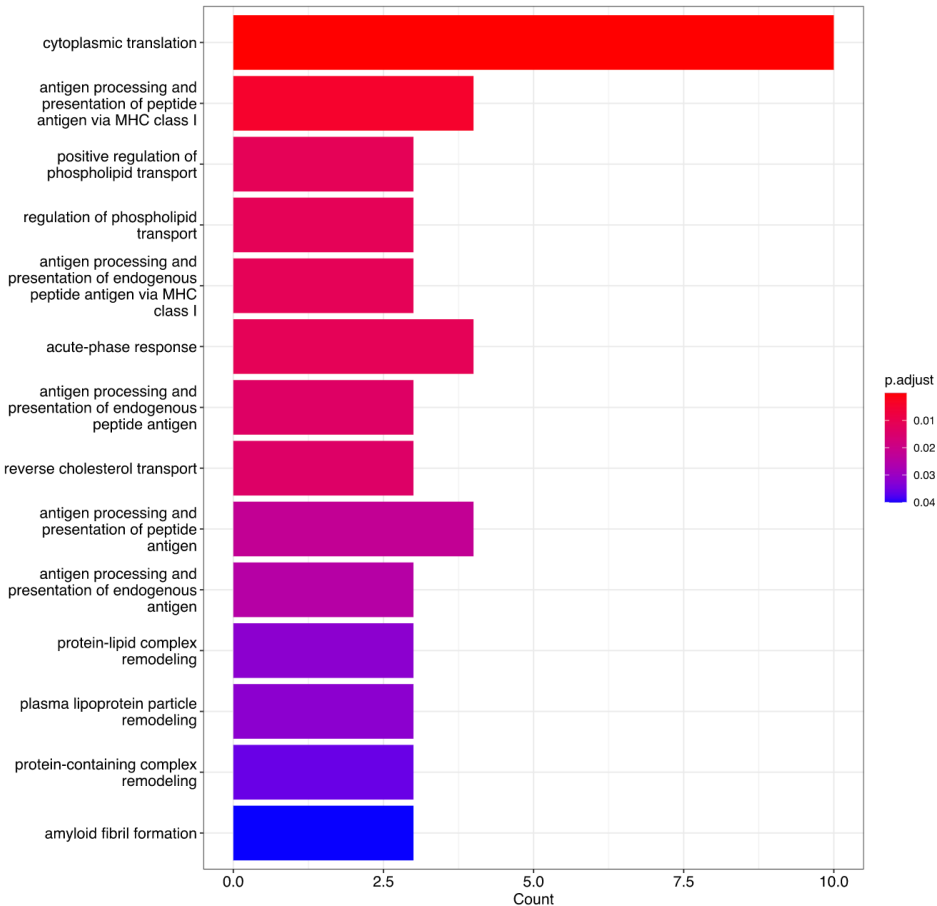

## b Immune cells

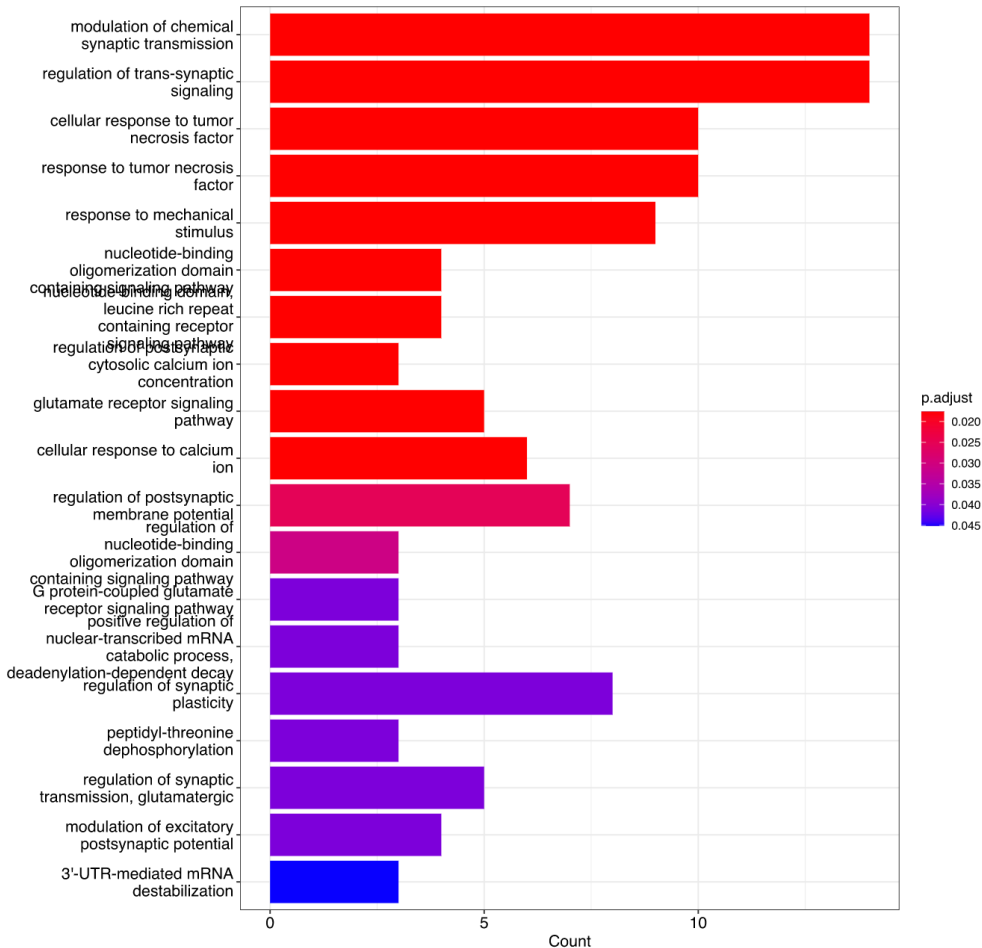

Supplement: Supplementary Figure 1 — Cohort characteristics of TNFA high and TNFA low groups in the blood and the intestine. (A) Cohort characteristics of patients with low (below the median) systemic expression of TNFA (n=22, black), and high (above the median) systemic expression of TNFA (n=22, red) in the blood are depicted. (B) Cohort characteristics of patients with low (below the median) intestinal expression of TNFA (n=22, black), and high (above the median) intestinal expression of TNFA (n=23, yellow) in the intestine are depicted. [file Presentation_1.pdf]
